# Mechanism of Inhibition of Enveloped Virus Membrane Fusion by the Antiviral Drug Arbidol

**DOI:** 10.1371/journal.pone.0015874

**Published:** 2011-01-25

**Authors:** Elodie Teissier, Giorgia Zandomeneghi, Antoine Loquet, Dimitri Lavillette, Jean-Pierre Lavergne, Roland Montserret, François-Loïc Cosset, Anja Böckmann, Beat H. Meier, François Penin, Eve-Isabelle Pécheur

**Affiliations:** 1 Institut de Biologie et Chimie des Protéines, UMR 5086, CNRS, Université de Lyon, IFR128 BioSciences Gerland-Lyon Sud, Lyon, France; 2 Physical Chemistry, ETH-Zurich, Zurich, Switzerland; 3 Université de Lyon, UCB-Lyon1, IFR128, Lyon, France; 4 INSERM, U758, Lyon, France; 5 Ecole Normale Supérieure de Lyon, Lyon, France; National Institute for Medical Research, Medical Research Council, London, United Kingdom

## Abstract

The broad-spectrum antiviral arbidol (Arb) inhibits cell entry of enveloped viruses by blocking viral fusion with host cell membrane. To better understand Arb mechanism of action, we investigated its interactions with phospholipids and membrane peptides. We demonstrate that Arb associates with phospholipids in the micromolar range. NMR reveals that Arb interacts with the polar head-group of phospholipid at the membrane interface. Fluorescence studies of interactions between Arb and either tryptophan derivatives or membrane peptides reconstituted into liposomes show that Arb interacts with tryptophan in the micromolar range. Interestingly, apparent binding affinities between lipids and tryptophan residues are comparable with those of Arb IC50 of the hepatitis C virus (HCV) membrane fusion. Since tryptophan residues of membrane proteins are known to bind preferentially at the membrane interface, these data suggest that Arb could increase the strength of virus glycoprotein's interactions with the membrane, due to a dual binding mode involving aromatic residues and phospholipids. The resulting complexation would inhibit the expected viral glycoprotein conformational changes required during the fusion process. Our findings pave the way towards the design of new drugs exhibiting Arb-like interfacial membrane binding properties to inhibit early steps of virus entry, i.e., attractive targets to combat viral infection.

## Introduction

**Figure 1:**
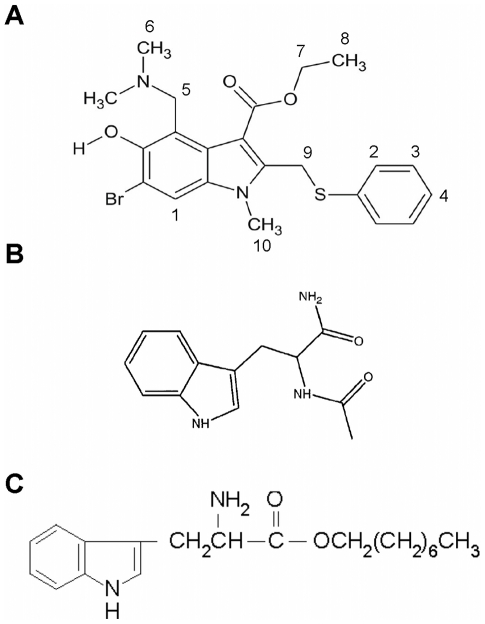
Chemical structures of arbidol (A), N-acetyl tryptophanamide (NATA) (B), and tryptophan octyl ester (TOE) (C). Note that numbering in panel A refers to proton numbers, as identified in NMR (cf Figure 5 and Table 1).

Distinct from specific antiviral compounds that target key viral functions are a group of broad-spectrum medicinal drugs that were originally designed for other treatments [1]–[3] or targeted toward a number of viruses ([4]; reviewed in [5]). The advantage of this group of antivirals is that they have already met the pharmacological criteria for medicinal drugs and are already approved for clinical use in some countries. Among these molecules, antiviral agents targeting viral entry of enveloped viruses are of major interest since they seize an early step in the viral life cycle, before damages have occurred to cells (recently reviewed in [6], [7]), and since they can be incorporated into combinations of multiple drugs with different targets. One of these compounds, arbidol [Arb; 1H-indole-3-carboxylic acid, 6-bromo-4-[(dimethylamino)-methyl]-5-hydroxy-1-methyl-2-[(phenylthio)methyl]-, ethyl ester, monohydrochloride; CAS Registry Number 131707-23-8 (Figure 1)], is already licensed in Russia and China, and is described as an anti-influenza drug with immunostimulant properties. Arb is in use for several years as prophylaxis and treatment for influenza A and B infections. It inhibits influenza virus-induced membrane fusion and may have the capacity to induce serum interferon [8]. Recent studies extended its inhibitory activity to other human viruses such as the respiratory syncytial virus, parainfluenza virus 3, rhinovirus 14, and hepatitis B virus (reviewed in [5], [9]). We demonstrated that it also inhibits hepatitis C virus (HCV) infection *in vitro*, and HCV replication [10], HCV cell entry and membrane fusion using HCV pseudoparticles (HCVpp) and HCV grown in cell culture (HCVcc) [11], [12]. Most recently, Ciliberto and coworkers demonstrated the efficacy of Arb derivatives at inhibiting HCV entry and replication into hepatoma cells in the low micromolar range [13]. HCV infection is a leading cause of liver diseases, including hepatocellular carcinoma, and therapeutic options are still limited (for recent reviews, see [14] and refs therein). There is thus an urgent need to develop efficient and well tolerated drugs to combat this virus.

Arb demonstrated a propensity to enter into hydrophobic interactions with membranes, and with membrane-like environments such as detergent micelles [12]. Here we further characterize the mechanism of action of arbidol, and analyze at the molecular and atomic level the interactions of Arb with membranes, tryptophan-rich derivatives and peptides. We first examined how Arb inhibits HCV entry and membrane fusion using HCVpp of different genotypes, and found that Arb inhibition was genotype-independent. By combining surface plasmon resonance, fluorescence and NMR spectroscopy approaches, we showed that Arb directly interacts with the phospholipid membrane interface, with an affinity in the micromolar range, comparable to the concentration inhibiting HCVpp membrane fusion by 50% (IC50). Arb also displayed micromolar affinity toward aromatic components of proteins such as tryptophan and derivatives, and toward peptides containing tryptophans and derived from HCV envelope glycoproteins. Altogether our results demonstrate that Arb interacts with the polar head of phospholipid membranes and protein motifs enriched in aromatic residues, suggesting that the inhibitory activity of Arb on HCV entry and fusion could involve both types of interactions.

## Materials and Methods

### Chemicals

Phosphatidylcholine from egg yolk (PC, 99% pure), dimyristoylphosphatidylcholine (DMPC, 99% pure), cholesterol (chol, 99% pure), lyso-phosphatidylcholine (lysoPC), dodecyl-phosphocholine (DPC), Triton X-100, tryptophan octyl ester hydrochloride (TOE) and *N*-acetyl-L-tryptophanamide (NATA) were purchased from Sigma. Octadecyl rhodamine B chloride (R_18_) was from Molecular Probes. The peptides used were part of the sequence of structural or non structural (NS) proteins of HCV and of the bovine viral diarrheal virus (BVDV). The amphiphilic helix of BVDV NS5A [15] and the transmembrane domain of HCV NS4A [16] were obtained as described previously. The peptides identified as important for HCV fusion [17] were purchased from Clonstar Biotech (90% purity) or Sigma Genosys (70% purity), respectively, and dissolved in DMSO before preparation of lipid∶peptide mixtures. Arbidol [Arb, 1H-indole-3-carboxylic acid, 6-bromo-4-[(dimethylamino)methyl]-5-hydroxy-1-methyl-2-[(phenylthio)methyl]-, ethyl ester, monohydrochloride (Figure 1)] was a kind gift from Stephen J. Polyak.

### Arb preparation

Arb was readily soluble in ethanol, and soluble in the mM range in water. Ethanol stock solutions of Arb were diluted to a 1.88 mM final concentration in milliQ water (the final stock solution contained 10% ethanol). For SPR experiments, one mg of Arb was resuspended in water, followed by centrifugation (16000×g, 15 min, 4°C). Arb concentration in solution was measured at 280 nm in the supernatant (Arb extinction coefficient = 9510 M^−1^.cm^−1^).

### Liposomes and micelles preparation

Mixtures of lipids [DMPC; PC; PC∶chol (70∶30, M∶M); PC∶chol∶R18 (65∶30∶5, molar)], of lipid∶peptide (20∶1, M∶M), of lipid∶TOE (20∶1, M∶M) or of detergent∶TOE (800∶1, M∶M) were prepared in chloroform∶methanol mixtures. After solvent evaporation, samples were resuspended in phosphate-buffered saline (PBS, pH 7.4) or water, and underwent 5 freeze/thaw cycles (liquid nitrogen and 37°C, respectively). Liposomes were prepared by extrusion over a stack of Avestin polycarbonate filters (100 nm), as described [18].

### Cell infection assays

Huh-7 cells [19] were maintained in DMEM containing 4.5 g/L d-glucose and 4 mM L-glutamine (Invitrogen, Cergy-Pontoise, France), supplemented with 100 U/ml penicillin, 100 µg/ml streptomycin and 10% FCS (Lonza). Productions of pseudotyped viruses were obtained by the transient transfection of 293T cells by the calcium-phosphate method. For the genotype study, HCVpp of genotypes 1a (H77; AF011752), 1b (Con1; AJ238799), 2a (JFH1; AB047639), 2b (UKN2B 2.8, AY734983), 3a (UKN3A 1.28, AY734984), 4a (UKN4 21.16, AY734987), 5a (UKN5.14.4, AY785283) and 6a (UKN6.5.340, AY736194) were produced as described previously [20] from 293T cells co-transfected with a murine leukemia virus (MLV) Gag-Pol packaging construct, an MLV-based transfer vector encoding GFP as a marker protein, and the E1–E2 expression constructs.

Supernatants were collected 48h post-transfection and filtered on 0.45 µm. For genotypes 5a and 6a, pseudoparticles were concentrated 100-fold after ultracentrifugation through a 20% sucrose cushion at 75,000×g for 2h at 4°C. Pellets were resuspended in the regular medium of Huh-7 cells. For infection experiments, Huh-7 cells were seeded at 4000 cells/well in 96-well plates. The following day, cells were infected in the presence of increasing Arb concentrations for 6 h. Arbidol effect on viral infectivity was evaluated by assaying GFP activity 72 hours after infection using flow cytometry (FACScalibur). Pseudoparticles harbouring at their surface the influenza hemagglutinin (HApp) and the envelope glycoprotein of the RD114 feline oncovirus (Rd114pp) were prepared as described in [18] and [12], respectively.

### Membrane fusion assays

Lipid mixing between pseudoparticles and PC∶chol∶R_18_ liposomes was monitored by fluorescent spectroscopy, as the dequenching of R_18_ [18]. In brief, R_18_-labeled liposomes (1 µl, 12.5 µM final lipid concentration) were added to a 37°C-thermostated cuvette containing pseudoparticles in PBS pH 7.4 [viral titers: H77 (1a) 5.10e^5^; W529A-HC 10e^2^; Con1 (1b) 4.10e^4^; JFH1 (2a) 5.10e^4^; AY734983 (2b) 8.10e^4^; AY734984 (3a) 3.10e^4^; AY734987 (4a) 9.10e^4^; AY785283 (5a) 3.10e^4^; AY736194 (6a) 5.10e^3^; HA 8.10e^8^; Rd114 2.10e^6^], and incubated 2 min. Fusion was initiated by acidification to pH 5 with HCl, and recorded on an SLM Aminco 8000 spectrofluorimeter over a 10-min time period, at excitation and emission wavelengths of 560 nm and 590 nm, respectively. Maximal R_18_ dequenching was measured after the addition of 0.1% Triton X-100 (final concentration) to the cuvette to lyse liposomes. The same procedure was used to follow pseudoparticle fusion in the presence of Arb; in this case, after a 1-min incubation of pseudoparticles with liposomes, Arb (11.3 µM final concentration) was added and incubated for 1 min, and fusion initiated by acidification.

### Fluorescence assays

Indole emission fluorescence spectra of tryptophan derivatives were recorded at excitation wavelength of 286 nm (spectral zone of lowest absorption of Arb), under various conditions: NATA (5 µM final) in PBS at pH 7.4 or 5.0; TOE at 5 µM in lyso-PC micelles (TOE∶lysoPC molar ratio 1∶800), and in PC and PC∶chol liposomes (TOE∶lipid molar ratio 1∶20); peptides at 5 µM in PC∶chol liposomes (peptide∶lipid molar ratio 1∶20). Spectra were obtained in the absence or presence of increasing concentrations of Arb (0 to 100 µM). Samples were incubated 2 min at 37°C prior to recording. Emission spectra were collected in the 300–400 nm region (with 2 nm-increments), with blanks substracted, using a black flat-bottom, low-binding 96-well microplate (Greiner Bio-one). Measurements were recorded on a Tecan Infinite® M1000 spectrofluorimeter. K_D_app values were calculated from the difference between the areas under the spectra in the absence or presence of Arb (ΔA), at various Arb concentrations, by nonlinear fitting using the equation ΔA = ΔA max C/(K_D_+C). Fluorescence measurements were repeated three times to obtain averaged values of K_D_app.

### Preparation of giant unilamellar liposomes

GUVs were made by the electroformation method [21]. The flow chamber (Warner Instruments, Connecticut, USA) used for vesicle preparation was equipped with two glass coverslips, each coated with optically transparent and electrically conductive indium tin oxide (ITO) (Philips, Eindhoven, NL). Mixtures of lipids [PC∶chol∶R_18_ (65∶30∶5, molar ratio)] were prepared at 0.1 mM in chloroform. The lipid mixture (2 nmoles) was spread into a thin and uniform film on the conductive face of ITO-coated slide. After chloroform evaporation, the dried lipid film was hydrated by adding water into the chamber (∼400 µl) and an alternative electrical field (10 Hz and 1.2V) was applied at room temperature for 3 hours. GUVs in the absence or presence of increasing amounts of Arb solubilized in water (0 to 40 nmoles), were observed by epifluorescence microscopy.

### Surface plasmon resonance (SPR)

Interaction of Arb with DMPC layers was investigated with a BIAcore 3000® using a L1 sensor chip at 30°C. The sensor chip surface was washed with a mixture of 50 mM NaOH and isopropanol (6∶4, v∶v), at a flow rate of 20 µl/min for 1 min. The running buffer was milliQ water. The influence of liposome concentrations on the final SPR signal was tested; we assayed lipid concentrations from 0.5 to 5 mM and measured the resulting resonance units (RU). We obtained a well detectable, reproducible and stable signal from 2 mM, and further increasing this concentration did not improve the signal. We therefore chose the 2 mM concentration for our experiments. DMPC liposomes were resuspended in milliQ water and captured on sensor chip at 2 µl/min for 5 min. The flow rate was increased to 30 µl/min and the liposome surface was then washed with 10 mM NaOH for 1 min. Liposomes immobilized on the chip surface gave approx. a 5000 RU signal. To calculate Arb affinity for lipids, its association to and dissociation from DMPC layers were studied at different Arb concentrations in water, from 0.5 to 10 µM, at a flow rate of 20 µl/min. After each binding cycle, the sensor surface was regenerated to the original matrix by injecting 50 mM NaOH/isopropanol (6∶4, v∶v). The sensor surface was then coated with a fresh liposome suspension for the next binding cycle. K_D_ values were calculated from the equilibrium resonance signal (R_eq_) as a function of the analyte concentration. R_eq_ values were estimated by extrapolation to infinite time using plots of resonance signal as a function of the reciprocal of time. Apparent K_D_ were then calculated by nonlinear fitting to the expression R_eq_ = R_max_C/(K_D_+C), where R_max_ is the maximum binding capacity of the surface and C is the analyte concentration, using the SigmaPlot software.

### NMR samples

For the NMR studies, the bicellar system was prepared by mixing 54 mg of 1,2-Dihexanoyl-sn-glycero-3-phosphocholine (DHPC) and 40 mg of DMPC with 400 µl of D_2_O. The sample with a lipid molar ratio [DMPC]/[DHPC] = 0.49 was subjected to 3 cycles of vortexing (2 min), heating to 313 K (20 min), vortexing (2 min) and cooling to 273 K (20 min). The clear lipid solution was then added to 2 mg Arb in powder, and then subjected again to the procedure of vortexing, heating, vortexing and cooling. The final molar ratio [Arb]/([DMPC]+[DHPC]) was 1/48, with [Arb] = 9.4 mM. Another sample with similar lipid concentration and higher Arb content (molar ratio 1/15) was also prepared. The amount of free Arb in Arb∶DMPC mixture was estimated after rapid separation of lipids on ultrafiltration membrane (cutoff 5000 Da) and measure of Arb concentration in the ultrafiltrate at 280 nm. For Arb∶DMPC molar ratio of 1∶4 at neutral pH, free Arb was found to be lower that 0.2%. We thus concluded that the amount of free Arb in NMR samples was negligible.

D_2_O from Cambridge Isotopes Lab (Cambridge, MA), and Gd(DTPA-BMA) was a generous gift of Klaus Zangger. All experiments were performed with freshly prepared samples.

### NMR spectroscopy

^1^H NMR experiments were performed on a Bruker DMX 400 spectrometer operating at a proton frequency of 400 MHz. Spectra were recorded with a 5 mm Triple Resonance Inverse TXI probe equipped with z-gradient. The π/2 pulse was 10.3 µs, the recycle delay was 3 s and solvent suppression with presaturation was used. 1D ^1^H spectra were measured acquiring 120 scans. These spectra were used for the assignment of the drug signals together with 2D NOESY and ^1^H/^13^C HSQC spectra (data not shown). The assignment of the lipid resonances was derived from the comparison with data in the literature [22]. To obtain Paramagnetic Relaxation Enhancements (PRE), a solution of Gd(DTPA-BMA) in D_2_O (60 mM) was added to the Arb/bicelle sample. Two sets of experiments were performed to measure the proton T1 relaxation times on the Arb/bicelle samples were performed. In the first one [Arb]/[Lipids] = 1/15, T = 305 K and [Gd(DTPA-BMA)] = 0, 2.0, 2.9, 4.6, 6.3, 7.9 and 9.7 mM. In the second one, [Arb]/[Lipids] = 1/48, T = 310 K and [Gd(DTPA-BMA)] = 0, 1, 2 mM. Proton T1 times were measured by using inversion recovery experiments with an inter-pulse delay ranging between 5 ms and 13 s. Each measurement was repeated 3 times, adding 240 scans with a delay time between scans of 15 s. All the spectra were processed using matNMR [23]. The ^1^H frequency scale is given in terms of chemical shift relative to the acetone signal used as an external reference (2.218 ppm).

## Results

### Differential arbidol inhibition of cell entry and membrane fusion of various HCVpp genotypes

**Figure 2:**
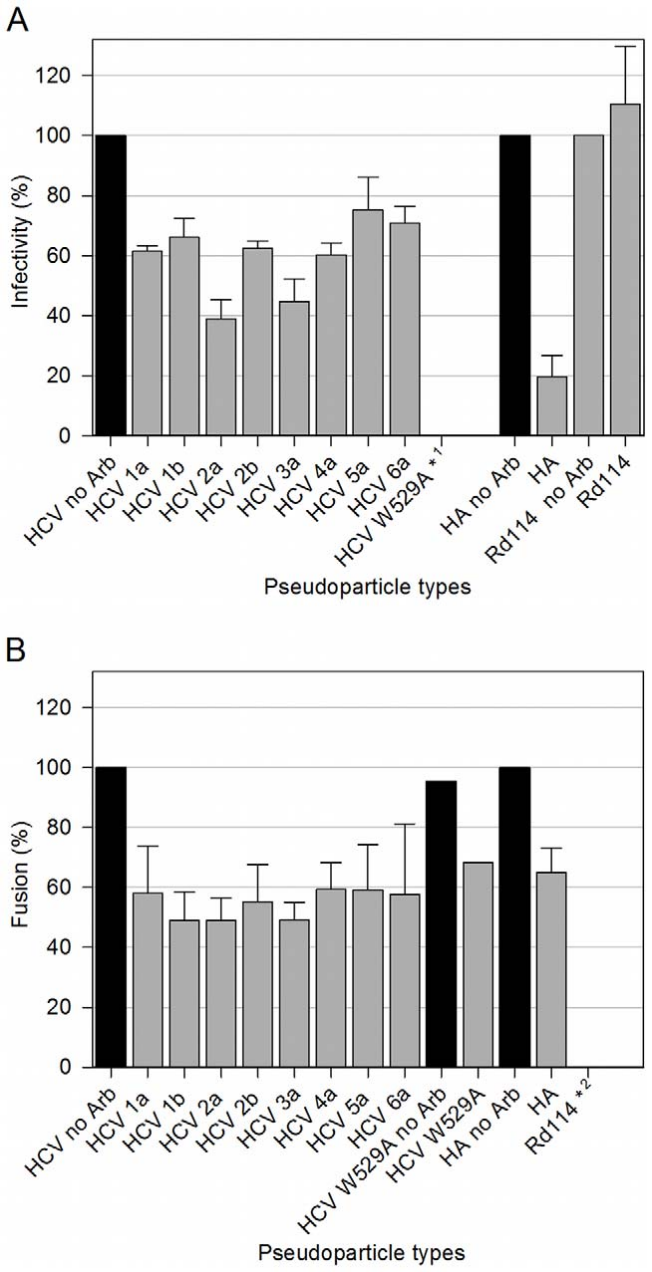
Arb inhibition of cell entry and membrane fusion of HCVpp of various genotypes. A, HCV entry assays using HCVpp in the absence or presence of 11.3 µM arbidol. Huh-7 cells were infected by co-incubating HCVpp of indicated genotype with or without Arb for 6 h. Infectivity was evaluated after 72 h by counting the percentage of GFP-positive cells, using a high-throughput flow cytometer (FACScalibur). The titer obtained in the absence of Arb was set to 100%, and the resulting percentages of infection in the presence of Arb were calculated. Results are the mean +/− SEM of 5 separate experiments. HApp are presented as control pseudoparticles sensitive to arbidol (cf also [10]), and Rd114pp insensitive to arbidol (cf also [12]). * ^1^, the mutant HCVpp W529A (cf [17]) are presented as a negative control of entry, displaying very low infectivity. B, Membrane fusion between HCVpp and R_18_-labeled liposomes was measured by recording the kinetics of lipid mixing by fluorescence spectroscopy (excitation and emission wavelengths were 560 and 590 nm, respectively), as described in the Materials and Methods section. Values of the last 30 s of fusion kinetics (final extent of fusion) were used to calculate the percentage of fusion in the presence of Arb, relative to fusion kinetics without Arb (100%). Results are the mean +/− SEM of 4 separate experiments. HApp and mutant HCVpp W529A were taken as controls. * ^2^: no fusion was observed for Rd114pp.

We have previously shown that Arb could inhibit cell entry and membrane fusion of HCVpp of genotypes 1a, 1b and 2a [10], [12], and HCVcc of genotype 2a [11]. Here we sought to investigate the effect of Arb on other major HCVpp genotypes as well. HCVpp infectivity toward Huh-7 cells, objectifying HCVpp entry, was assayed by counting cells positive for GFP (as the marker protein), incubated with or without increasing Arb concentrations for 6h (see Materials and Methods). A representative data set is shown in Figure S1A, and inhibition obtained for the highest concentration of Arb (11.3 µM) is presented in Figure 2A. The inhibitory effect of Arb on HCVpp cell entry depends on HCVpp genotype. Indeed, within biological intrinsic variability of HCVpp preparation and samples, three cases could be distinguished: entry of HCVpp of genotypes 2a and 3a was inhibited by ca. 60%, while 1a, 1b and 2b exhibited a 40%-inhibition, but entry of genotypes 5a and 6a was weakly affected by the presence of Arb (Fig. 2A). The influence of Arb on HCVpp-mediated lipid mixing was assayed by fluorescence spectroscopy using fluorescent liposomes, as previously described [18]. Lipid mixing between HCVpp and liposomes was only observed at low pH and optimal at pH 5.0 [18]. In the presence of increasing Arb concentrations, lipid mixing was inhibited in an Arb dose-dependent manner (Figure S1B for HCVpp genotype 4a). In contrast to what was observed for HCVpp infectivity, the effect of 11.3 µM Arb on HCVpp-mediated membrane fusion (Figure 2B) was similar for all tested genotypes, with about 50% inhibition of membrane fusion activity. This indicates that membrane fusion inhibition by Arb is not genotype-dependent.

These data suggest that the differential inhibitory effect of Arb on HCVpp infectivity of various genotypes is likely due to a genotype-dependent modulation of HCV glycoproteins interaction with the *cellular proteins* (e.g. HCV receptors) involved in HCV cell entry. Conversely, Arb inhibition of HCVpp membrane fusion, as assessed by a *in vitro* model system where the only proteins present are the viral glycoproteins, could merely reflect the interaction of Arb on lipids and/or on motifs present in HCV glycoproteins of any genotype. To test these hypotheses, we further investigated Arb interaction properties with lipids and protein fragments using the approaches described in the following.

### Arbidol interactions with lipid membranes

**Figure 3:**
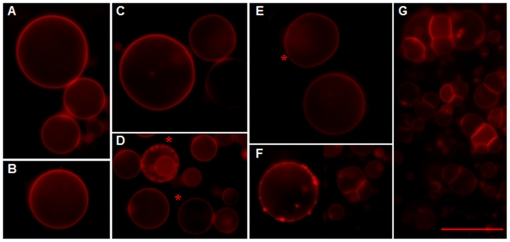
Arb interacts with lipid bilayers of giant unilamellar liposomes. GUV composed of PC∶chol∶R_18_ (2 nmol) were electroformed in water and observed by optical epi-fluorescence microscopy (A). Various concentrations of Arb in water were added to GUV, for final Arb-to-lipid molar ratios of: B, 1∶40; C, 1∶20; D, 1∶10; E, 1∶1; F, 10∶1 and G. 20∶1. Asterisks indicate small invaginations (panel D) or occasional GUV flickering (panel E). Bar, 25 µm.

We previously showed that Arb could interact with liposomes and membrane-like environments such as detergent micelles [12]. We further investigated this feature by studying the interactions of Arb with giant unilamellar liposomes (GUV) by optical microscopy (Figure 3). GUV are pure lipid bilayers, intrinsically flexible and unstable due to their very large size (in the range of tens of µm) [24]. Increasing Arb concentrations were added to the chamber where GUV composed of PC∶chol were electroformed (see Methods section), with Arb-to-lipid molar ratios of 1∶40, 1∶20, 1∶10, 1∶1, 10∶1 and 20∶1. The GUV bilayer was unaffected by the presence of Arb up to a 1∶20 Arb-to-lipid ratio, with occasional membrane flickerings (Fig. 3C and asterisk in Fig. 3E). At higher ratios, membrane inhomogeneities and invaginations appeared (Fig. 3F, asterisks in Fig. 3D), and a major overall membrane reorganization was observed at a 20∶1 Arb-to-lipid ratio (Fig. 3G). Note that no lysis or membrane dislocation of GUV was observed for any condition, even at the highest ratio (data not shown). These results reveal that only very high concentrations of Arb with respect to lipids could significantly perturb the lipid organization of these bilayers. This also indicates that the direct interaction of Arb to lipid bilayers at the concentrations used to inhibit HCVpp infectivity and membrane fusion (panel E) do not perturb lipid organization.

In addition, HCVpp pre-incubated at neutral or acidic pH with Arb, even at very high concentrations (100 µM), displayed similar morphology (visualized by transmission electron microscopy) as those observed in the absence of the drug (data not shown). Indeed we counted over 160 HCVpp for each condition, and no difference in HCVpp morphology could be observed between the parameters assessed. This indicates that Arb inhibition of HCVpp fusion is not due to viral particle disruption/damage.

### Surface plasmon resonance

To gain insight into the molecular details of the interaction of Arb with lipid membranes, we next investigated the lipid binding properties of Arb by using surface plasmon resonance (SPR, Biacore® technology). We used a Biacore's L1 sensor chip to capture DMPC liposomes. This sensor chip displays lipophilic groups attached on the surface of a carboxymethylated dextran layer, and was shown to provide a quick and reproducible method for the preparation of bilayer-mimetic systems [25]. We first tested whether arbidol *per se* could bind or not to the chip. Arb at 11.3 µM (the highest concentration relevant in the biological context) was injected onto the chip devoid of liposomes. This led to approx. 60 resonance units (RU, see Methods section). DMPC liposomes (2 mM) captured onto the sensor chip reached about 5000 RU, and a further ∼600 RU was seen when Arb was pulsed onto the liposome-coated chip. The binding of arbidol alone on the L1 chip remains therefore negligible.

**Figure 4:**
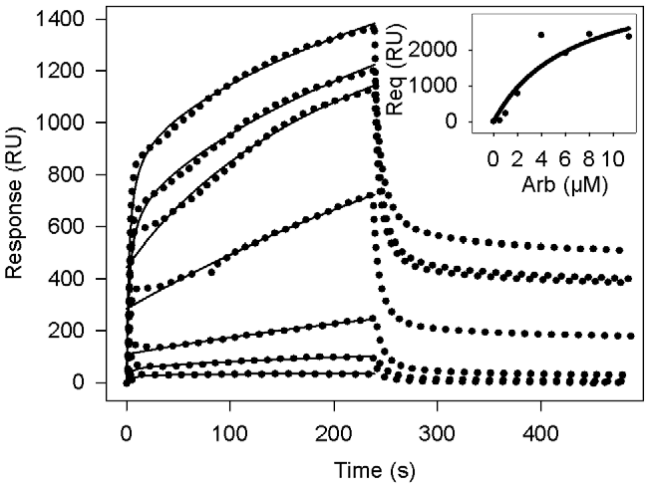
Binding of Arb to immobilized DMPC membranes. Arb in water at concentrations of 0.5, 1, 2, 4, 6, 8 and 11.3 µM was injected over immobilized DMPC membranes (ca. 5000 resonance units) for 4 min at a flow rate of 20 µl/min, followed by water. Blank curves without Arb were substracted from those obtained with Arb. Inset, representative set of data of non-linear regression fits to the equilibrium resonance signal (R_eq_), obtained by extrapolation to infinite time (see Materials and Methods), *vs* Arb concentration, used to obtain apparent equilibrium dissociation constant (K_D_) as well as the maximum binding capacities (R_max_). Kinetics were reproduced 4 times. Dotted curves represent the sensorgram and solid curves the non-linear fit. RU, resonance units.

Measures of Arb/DMPC association and dissociation were performed with various Arb concentrations ranging from 0.5 to 11.3 µM. After passage over the surface of the sensor chip, Arb bound to immobilized DMPC in a concentration-dependent manner (Figure 4). Arb initial binding was fast, but then slowed down without reaching saturation equilibrium (from 0 to 240 s). After stopping the Arb flow onto the sensor chip (from 240 s), bound Arb was rapidly but incompletely dissociated from DMPC membranes. Indeed, for all Arb concentrations tested, about 50% of Arb remained bound to DMPC. This demonstrates that Arb is capable of interacting with lipid membranes, in a stable association between Arb and DMPC. However the behaviour of Arb binding to membranes rendered difficult the fitting of a kinetic model to the data, and hence the determination of reliable on- and off-rates. Indeed using global fitting, binding curves could not be fitted properly with the different models included in the BIAevalution 3.0 software (1∶1 Langmuir binding, bivalent analyte, heterogeneous ligand, heterogeneous analyte, conformational change), with or without mass transport. Furthermore, because equilibrium was not reached during the association phase, the direct use of Scatchard analysis to calculate the apparent equilibrium dissociation constant was not allowed. Instead, the apparent equilibrium dissociation constant K_D_ was calculated from the equilibrium resonance signal (R_eq_) as a function of analyte concentration, R_eq_ values being estimated by extrapolation to infinite time using plots of resonance signal as a function of the reciprocal of time [26], [27]. Apparent K_D_ was then calculated by nonlinear fitting to the expression R_eq_ = R_max_C/(K_D_+C), where R_max_ is the maximum binding capacity of the surface and C is the analyte concentration, using SigmaPlot software. This calculation, performed on 4 separate experiments, gave an apparent K_D_ of 6.8±0.4 µM (see inset to Figure 4 for a representative experiment). This dissociation constant is in the same order as the IC50 of HCVpp fusion (11.3 µM). This result indicates that the inhibitory effect of Arb on HCVpp membrane fusion is at least in part deriving from Arb association to lipid membranes.

### NMR experiments

**Figure 5:**
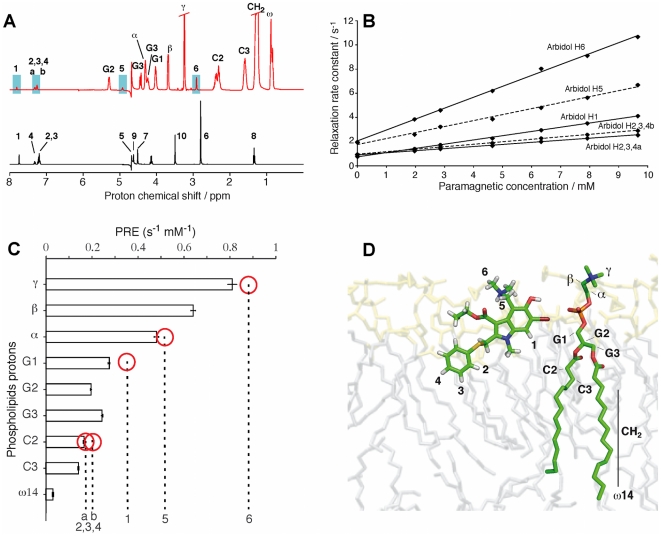
NMR of Arb into lipid bicelles. A, ^1^H NMR spectrum of Arb in D_2_O (in black) and in DMPC/DHPC bicelles (in red) with [Arb]/[lipids] = 1/15 and T = 305 K. B, influence of the concentration of the paramagnetic agent Gd(DTPA-BMA) on the proton relaxation times for Arb in the bicelle system. C, paramagnetic relaxation enhancements (PRE) measured on Arb (marked by dotted vertical lines) and on the phospholipid protons (marked by histogram bars). The phospholipid is used as a yardstick to roughly estimate the arbidol proton positions inside the membrane. Red circles indicate the yardstick marker closest to a given arbidol PRE value. Error bars indicate the standard deviation derived from the calculation of PRE. Error bars for Arbidol are comparable. D, sketch of the positioning of Arb in a DMPC membrane system. The Arb molecule was produced by generating an extended structure, and regularized by 1000 cycles of a Powell type minimization using XPLOR-NIH [69]. The positioning in the membrane system was done manually by taking into account the relative proton depth measured by the PRE (panel C).

NMR spectroscopy was used to characterize the Arb insertion in a model membrane system. The ^1^H NMR spectrum of Arb recorded at 305 K in deuterated water is shown in Figure 5A (black spectrum), and the assignment of the proton signals deduced from 2D spectra analysis (data not shown) are reported in Table 1. In order to study Arb in a membrane-mimetic environment, we used isotropic phospholipid bicelles consisting of a mixture of DMPC/DHPC in water. Long-chain phospholipid molecules of DMPC self-assemble into planar bilayers, while the short-chain molecules of DHPC segregate to edge regions of high curvature [28]. Bicelles with [DMPC]/[DHPC] molar ratio <1 form fast and isotropically tumbling aggregates, amenable to solution NMR studies. Still, isotropic bicelle systems are used as a phospholipid bilayer mimetic, since DMPC has been shown to form a flat bilayered surface [29]–[31]. The ^1^H NMR spectrum of Arb in this bicellar phase is shown in Figure 5A (red spectrum). In this system, the spectral crowding due to the presence of phospholipid resonances allowed only the observation of protons denoted 1, 2, 3, 4, 5 and 6 of the arbidol molecule (see Fig. 1A) where only 2 signals can be distinguished for the three protons 2, 3, 4. Additional ^1^H resonances could be resolved from 2D ^1^H NOESY spectra and ^1^H/^13^C HSQC spectra (Figure S2) and the corresponding chemical shifts are shown in Table 1 together with the assignment of the proton lines for Arb in water. Arbidol interaction with lipids induce chemical-shift changes in the Arb resonances when compared to that observed in water.

**Table 1:** NMR assignment of ^1^H chemical shift of Arb in water and in the presence of [DMPC]/[DHPC] bicelles at 305 K.

Arb protons	Arb in water (ppm)	Arb in bicelles (ppm)
1	7.75	7.81
02/04/10	7.18–7.34	7.25–7.33
5	4.63	4.93
6	2.8	2.92
7	4.15	*nd*
8	1.35	*nd*
9	4.52	4.68
10	3.5	3.67

*nd*, protons 7 and 8 could not be resolved from the lipid resonances (see Figure S2). Protons 9 and 10 can be resolved only in the ^1^H/^13^C HSQC spectrum.

In order to investigate the immersion depth of Arb in the membrane, we monitored the proton longitudinal relaxation rate of Arb protons upon the addition of the soluble paramagnetic agent gadolinium-diethylenetriamine pentaacetic acid-bismethylamide Gd(DTPA-BMA) [32]. This paramagnetic contrast agent stays soluble in the water surrounding the membrane and induces a paramagnetic relaxation enhancement (PRE) on the spin of the atoms close to the surface of the membrane. Recently, PRE effects due to Gd(DTPA-BMA) were used to probe the immersion depth and orientation of an anti-microbial peptide [33], [34]. Here we measured the proton T_1_ relaxation times of both Arb and phospholipid protons to probe the immersion depth of Arb in the membrane, using the PRE values of the phospholipids as an approximated yardstick. A titration of Arb with Gd(DTPA-BMA) was performed at increasing concentrations of the paramagnetic agent, from 2.0 to 9.7 mM. At each step of the titration, the proton *T*1 relaxation times for the protons 1, 2, 3, 4, 5 and 6 of Arb, and for the protons alpha, beta, gamma, G1, G2, G3, C2, C3 and omega14 of the phospholipids, were measured. The plot in Figure 5B shows the relaxation times as a function of the Gd(DTPA-BMA) concentration. This titration leads to a curve for each proton whose slope corresponds to the PRE values. As shown in Fig. 5C, PRE measured on the Arb-bicelle system range from 0.90 sec^−1^ to 0.03 sec^−1^ for the phospholipid protons and from 0.90 sec^−1^ to 0.20 sec^−1^ for Arb protons. The PRE observed for each spin can be described as an overall relaxation enhancement [35], due to all the paramagnetic agents in solution. For a planar membrane surrounded by a buffer containing a non-interacting paramagnetic probe, the total PRE of a nucleus with immersion depth *d* is given by the equation [33], [34]: PRE = *z*/*d* ^3^, where *d* is the immersion depth of a specific nucleus within the membrane *plus* the radius of the magnetic probe, and where the constant *z* is a combination of various parameters, among them a correlation time, itself a combination of the electron relaxation time, the lifetime of the intermolecular adduct bicelle-Gd(DTPA-BMA), and the rotational correlation time. In order to determine the immersion depth of Arb, instead of determining *z*, we used the phospholipids as a yardstick by comparing their PRE with the one of Arb.

The PREs of the resolved signals of Arb and phospholipids are reported and compared in Figure 5C. This procedure is based on two assumptions: (i) the amount of free Arb in solution in the presence of lipids is negligible (see NMR sample preparation in Experimental Procedure section), and it does therefore not affect significantly the PRE of Arb in the membrane ; ii) the constant *z* is the same for lipids and Arb.

Figure 5C shows that methyl protons 6 of Arb are at the lipid/water interface as the gamma-proton of the hydrophilic head-group of the lipids. Protons 5 and 1 of Arb are at the level of respectively protons alpha and G1 of the phospholipids. The aromatic protons 2 and 3 are the most buried protons of Arb, close to the beginning of the hydrophobic chain (protons C2).

The validity of assumption (ii) is supported by the NOE cross-peaks detected between proton 1 of Arb and the glycerol moiety of phospholipids. In addition, the maximum PRE measured for Arb (protons 6) and phospholipids (γ protons) are about the same, suggesting that the corresponding molecule regions are the most exposed to water. This estimation of the immersion depth of Arb protons relative to the phospholipids protons enables us to propose a model for the positioning of Arb in the membrane, shown in Figure 5D.

These NMR data clearly demonstrate that Arb interacts at the membrane interface, mainly at the level of phospholipid polar head. This result supports the assumption that the Arb inhibitory effect on HCVpp membrane fusion is dependent, at least in part, from this interaction.

### Arbidol interaction with tryptophan derivatives and protein motifs

A second possibility regarding Arb activity is that Arb might interact with key motifs present in viral proteins, thereby impeding their structural reorganization at the onset of fusion and thus leading to fusion inhibition.

A first set of experiments was designed to investigate whether the order of addition of fusing partners would affect Arb-induced fusion inhibition. For this purpose, we measured fusion after pre-incubation of HCVpp or liposomes or both in the absence or presence of 11.3 µM Arb. As shown in Table 2, when Arb was pre-incubated with both partners before fusion was initiated by lowering the pH, fusion inhibition was ca. 50%. In contrast, only ca. 10% fusion inhibition was observed when Arb was pre-incubated with either HCVpp or liposomes. The greater inhibitory effect of Arb when it has simultaneously access to both viral and target membranes suggests that Arb could also act by interacting with selective residues of the HCV glycoprotein sequences.

**Table 2:** Influence of the order of addition of fusing partners on HCVpp 1a fusion inhibition by Arb.

Pre-incubation conditions				
Component	Time (min)	Component	Time (min)	Inhibition of fusion with Arb^a^ (%)
HCVpp/liposomes	2	-	-	46±12
HCVpp	1	Liposomes	1	5±7
Liposomes	1	HCVpp	1	13±11

a Arb (11.3 µM) was pre-incubated sequentially with either HCVpp or R_18_-labeled liposomes or both for the indicated times at 37°C before initiating lipid mixing by decreasing pH to 5.0 as described in the legend to Figure 2. The extent of inhibition of fusion by Arb was calculated relatively to the fusion observed in the absence of Arb and normalized to 100%. Results are the mean ± SEM of 3 separate experiments.

**Figure 6:**
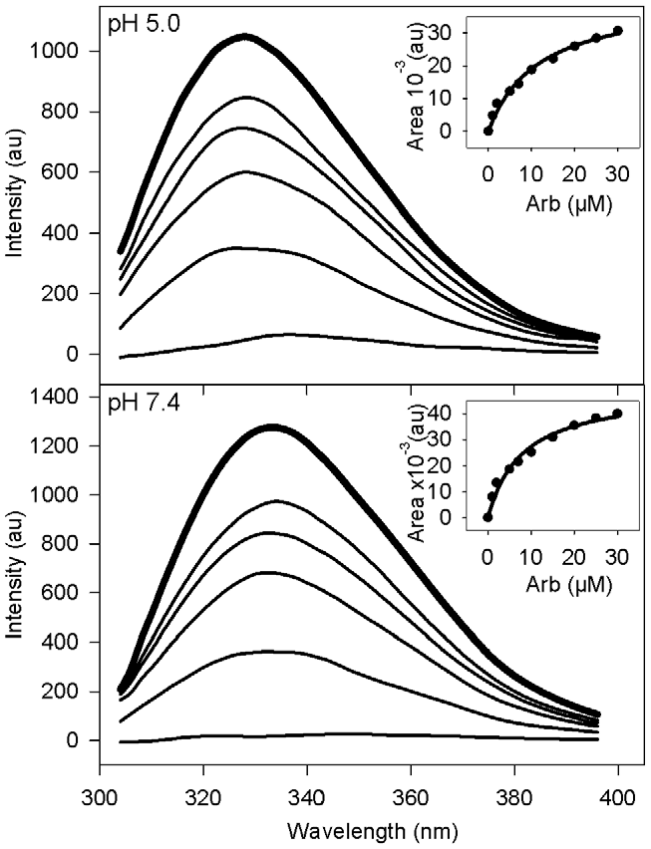
Indole emission fluorescence spectrum of TOE into PC∶chol liposomes. PC∶chol (70∶30 molar ratio) liposomes containing TOE (5 µM final, lipid-to-TOE ratio 20∶1) were equilibrated to 37°C in PBS at pH 7.4 or 5.0, in the absence (bold line) or presence (standard lines) of increasing concentrations of Arb (2, 5, 10, 25 and 100 µM). Indole fluorescence was measured between 300 and 400 nm, with excitation at 286 nm. The apparent affinity of Arb toward TOE was calculated from the plot of the difference ΔA between spectral areas (AUC) of TOE without or with Arb (ΔA = AUC_no Arb_−AUC_with Arb_) as a function of Arb concentration (see inset for a range of Arb concentrations between 0 and 30 µM) (see K_D_ values reported in Table 3).

This assumption was tested by studying the interaction behaviour of Arb with tryptophan (Trp) derivatives, as tryptophan is a constituent of proteins often found in regions located close to membrane interfaces, such as stem regions in several viral fusion proteins (e.g. HIV gp41 [36]). We also reasoned that Arb, being an indole derivative, might interact with tryptophan and tyrosine residues through aromatic ring stacking. For this purpose, we tested the effect of increasing concentrations of Arb on the fluorescence of N-acetyl tryptophanamide (NATA) as a water-soluble Trp derivative, and tryptophan octyl-ester (TOE) as a membranotropic molecule (Figure 1B–C). The fluorescence of NATA and TOE was recorded between 300 and 400 nm, using an excitation wavelength of 286 nm, which corresponds to an absorption minimum of Arb [12]. Results are presented in Table 3 and Figure 6. The apparent affinity of Arb for indole of Trp derivatives was calculated as the difference (ΔA) between spectral areas (AUC) in the absence and presence of Arb (i.e. ΔA = AUC_no Arb_–AUC_Arb_), for each Arb concentration at pH 7.4 or 5.0. Apparent K_D_ values were then calculated from the plots ΔA = f([Arb]), by non-linear fitting. Apparent K_D_ of Arb for NATA in solution was found in the range of ca. 60 µM at both pH (Table 3). Similarly, the K_D_ of Arb for TOE in lyso-PC micelles was in the 50 µM range at both pH. This indicates that Arb is able to interact with indole rings, but with a relatively low affinity. NMR analysis of Arb in dodecyl-phosphocholine (DPC) micelles in the presence of TOE further confirmed this interaction (TOE/Arb/DPC molar ratios: 1∶0.5∶50; 1∶1∶50; 1∶2∶50 and 1∶5∶50). Indeed, chemical shift variations of aromatic protons from Arb and TOE were observed when comparing the NMR spectra of Arb/DPC, TOE/DPC, and Arb/TOE/DPC samples (data not shown).

**Table 3:** Apparent dissociation constants between Arb and the indole ring of the tryptophan derivatives NATA and TOE.

	K_D_ (µM)^a^	
Tryptophan derivatives and media	pH 7.4	pH 5.0
NATA in solution	64±10	55±10
TOE in lyso-PC micelles^b^	58±9	48±4
TOE in PC liposomes^c^	6.0±0.5	9.2±0.2
TOE in PC∶chol liposomes^c^	11.7±1.4	15.6±1.3

a For experimental details, see legend to Figure 6.

b TOE-to lyso-PC molar ratio was 1∶800.

c TOE-to-lipid molar ratio was 1∶20.

In contrast, when Arb was added to TOE associated to liposomes (1∶20, TOE/lipid molar ratio), a marked increase in affinity was observed (Table 3, compare TOE/micelles and TOE/liposomes), reaching K_D_ values in the 10 µM range. Note that TOE fluorescence could not be measured in DPC micelles, due to a great intrinsic fluorescence of the DPC used for our experiments. Interestingly, these K_D_ values are comparable to Arb IC50 inhibition of HCVpp fusion (see above and Discussion section). Indole fluorescence decreased when Arb concentration increased, with virtually no measurable fluorescence for 100 µM Arb (Figure 6). This further confirms that Arb interacts with indole rings, but with a higher affinity when indole is incorporated into lipid membranes. This affinity was higher for PC than for PC∶chol liposomes (Table 3), and at neutral than at acidic pH (Table 3 and Figure 6). At acidic pH, Arb is most likely protonated in the lipid environment [12], as is probably TOE as well. Arb affinity for TOE under these conditions might then be lower than that of uncharged Arb at neutral pH, because of repulsive electrostatic interactions.

**Figure 7:**
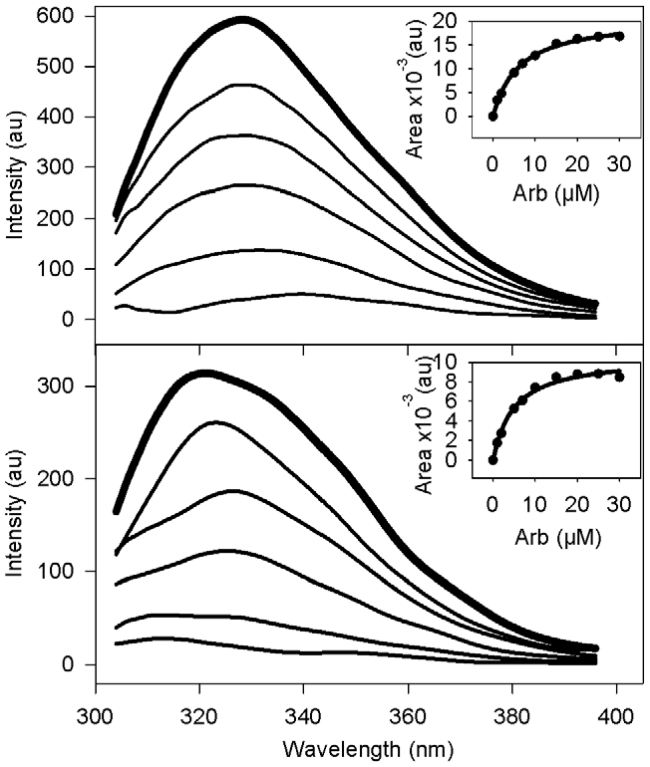
Trp emission fluorescence spectrum of an HCV model peptide into PC∶chol liposomes. PC∶chol (70∶30 molar ratio) liposomes containing HCV NS4A peptide (KKGGSTWVLVGGVLAALAAYCLSTGSGGKK, 5 µM final, lipid-to-peptide ratio 20∶1) were equilibrated to 37°C in PBS at pH 7.4 or 5.0, in the absence (bold line) or presence (standard lines) of increasing concentrations of Arb (2, 5, 10, 25 and 100 µM). Trp fluorescence was measured between 300 and 400 nm, with excitation at 286 nm. The apparent affinity of Arb toward Trp was calculated from the plot of the difference ΔA between areas under the curve (AUC) of peptide without or with Arb (ΔA = AUC_no Arb_−AUC_with Arb_) as a function of Arb concentration (see inset for a range of Arb concentrations between 0 and 30 µM) (see K_D_ values reported in Table 4).

A third set of experiments was designed to assess the behaviour of Arb in the presence of aromatic residues into protein sequences, more specifically toward Trp present in peptides. For this purpose, we used synthetic membrane-binding peptides of known structure and containing only one tryptophan residue, expected to be localized at the membrane interface: the transmembrane helix of HCV NS4A protein [16] and the N-terminal amphipathic helix of BVDV NS5A protein interacting in-plane of the membrane interface [15]. Intrinsic tryptophan fluorescence of both peptides was monitored in the presence of increasing concentrations of Arb; this is illustrated in Figure 7 for HCV NS4A peptide inserted into PC∶chol liposomes (and in Figure S3 for BVDV NS5A, 1∶20 peptide-to-lipid molar ratio). The Arb dose-dependent quenching of tryptophan fluorescence at both neutral and acidic pH clearly indicates Arb interaction with both peptides (insets in Figure 7 and Fig. S3). For the NS4A peptide at both pH, a red shift of the spectral maximum, proportional to Arb concentration, accompanied the fluorescence quenching; this effect was more pronounced at acidic pH (6 nm at pH 7.4 for 5 µM Arb, and 12 nm at pH 5.0 for 100 µM Arb). This red shift suggests that Arb, when interacting with NS4A peptide, relocates its tryptophan residue to a more shallow zone of the membrane, where the Trp environment would be more hydrophilic. The measure of the apparent affinity of Arb for these peptides inserted into PC∶chol liposomes was performed as described above. Interestingly Arb displayed an apparent K_D_ toward peptide Trp between 3.3 and 5.6 µM (Table 4), twice lower than that observed for TOE in PC∶chol liposomes. Since both peptides contain one or two tyrosine residues (HCV NS4A and BVDV NS5A, respectively) in addition to the Trp, interaction of Arb molecules with these aromatic residues might account for a higher affinity of Arb for peptides than for a small molecule such as TOE.

**Table 4:** Apparent dissociation constants between Arb and Trp residues of model membrane-binding peptides inserted into liposomes.

	K_D_ (µM)^c^	
Membrane peptides^a^ inserted in PC∶chol liposomes^b^	pH 7.4	pH 5.0
HCV NS4A[1–22]* transmembrane peptide *KKGG*STWVLVGGVLAALAAYCLSTGS*GGKK*	3.3±0.6	5.6±0.3
BVDV NS5A[1–28] membrane anchor peptide SGN**Y**VLDLI**Y**SLHKQINRGLKKIVLG**W**A	3.3±0.4	4.1±0.4

a The NMR structures of synthetic peptides HCV NS4A[1–22]* and BVDV NS5A[1–28] peptides have been reported in references [16] and [15], respectively. The solubilization tags KKGG and GGKK at the N- and C-terminal ends, are indicated in italic. Aromatic residues Trp and Tyr are indicated in bold, His is underlined.

b Peptide-to-lipid molar ratio was 1∶20.

c K_D_ values were calculated as described in legend to Figure 7.

Since Arb is an inhibitor of HCV membrane fusion, we reasoned that it might interact with the regions of E1 and E2 described as important for HCV fusion [11], [17]. These peptides were described as membranotropic on model membranes [37] and contain aromatic residues. We therefore analyzed the effect of increasing concentrations of Arb on the fluorescence quenching of two peptide sequences derived from HCV E2 (positions 415–432 and 606–625, see aa sequences in Table 5), and inserted into PC∶chol liposomes (1∶20 peptide-to-lipid molar ratio). Note that we also tested a third peptide located at position 270–283 of E1, containing only one Tyr; but its fluorescence quantum yield was too low to monitor any interpretable fluorescence signal (data not shown). We then calculated the K_D_ values as described above. As shown in Table 5, E2 415–432 contains only one Trp, whereas E2 606–625 contains one Trp and 3 Tyr. The apparent affinity was in the 15 µM range at pH 7.4 for both peptides, reminiscent to Arb IC50 of HCVpp fusion. This indicates that Arb is able to interact with the aromatic residues of both peptides in the membrane, and lends further support to our hypothesis that Arb could interact with key residues/motifs in viral fusion proteins, which would constitute a possible (partial) explanation to its inhibition of HCVpp fusion. Strikingly this affinity decreased at acidic pH for both peptides, and even drastically to 70 µM for E2 606–625. Interestingly, this relatively high K_D_ value is reminiscent of that observed for Arb interaction with NATA in solution (Table 3). This suggests that the interaction between the E2 peptide and the membrane would be weak at acidic pH, and that most of the peptide could be in solution. Moreover an histidine residue, located in the immediate vicinity of Trp in the sequence of both peptides, is expected to be charged at pH 5.0. Since Arb is also protonated at that pH value, this could create repulsive forces affecting the interaction between Trp and Arb. Moreover, as protonation of the histidine cycle is expected to decrease the free energy of partition from lipids to water, the peptide could be released from the membrane at acidic pH, possibly in relation with peptide conformational change(s). This behavior in not in favor with their direct role as fusion peptides of HCV, a virus dependent on low pH for its membrane fusion activity. However, due to their membranotropism [37], and since our and other studies showed their involvement in HCV membrane fusion [17], [38], it is possible that the conformational changes they might undergo at low pH would lead to a proper relocation of the actual fusion peptide/loop toward the target membrane [39] (and see Discussion section).

**Table 5:** Apparent dissociation constants between Arb and Trp residues of synthetic peptides involved in fusion.

	K_D_ (µM)^c^	
Membrane peptides^a^ inserted in PC∶chol liposomes^b^	pH 7.4	pH 5.0
E2[415–432] NTNGS**W** HINSTALNCNES	15±4	28±7
E2[606–625] RCMVD**Y**P**Y**RL**W** H **Y**PCTIN**Y**T	13±3	70±10

a Peptides from HCV strain H (genotype 1a, accession number AF009606; [20]). Aromatic residues Trp and Tyr are indicated in bold, His is underlined.

b Peptide-to-lipid molar ratio was 1∶20.

c K_D_ values were calculated as described in legend to Figure 7.

## Discussion

This study aimed at further investigating the molecular mechanism of action by which arbidol (Arb) inhibits virus cell entry and membrane fusion, using HCVpp as a model of an enveloped virus.

We showed that Arb displayed a dual binding capacity, on lipid membranes interface on one hand and on the aromatic residue tryptophan of proteins on the other hand. It therefore appears plausible that the observed inhibitory effect of Arb on viral entry and membrane fusion might result from a combined effect of binding of Arb on membranes and on (fusion) proteins.

From a physico-chemical point of view, Arb displayed tropism for membranes or membrane-like environments such as detergent micelles, particularly prominent at low pH [12]. By combining several biochemical approaches, we show here that Arb has the propensity to bind to and incorporate into lipid bilayers, with calculated apparent affinities in a similar range as the IC50 value for fusion, i.e. ca. 10 µM. Our NMR studies of Arb interaction with DMPC leads to a model where Arb binds at the membrane interface and establishes contacts mainly with the polar heads of phospholipids (Fig. 5D).

Altogether these data suggest that at least part of Arb inhibitory activity could be explained by its membranotropism. This physico-chemical property has been further emphasized in a recent work by Villalain [40], using Fourier-transform infrared spectroscopy. Arb interaction with phospholipids would disturb membrane fluidity crucial to the fusion process, thereby rendering the lipid bilayer less prone to fusion. Such a model is consistent with the behavior of other indole derivatives, that were shown to exhibit a preference for membrane interfaces [41], [42], due to the flat rigid structure of these molecules and to their aromaticity, which allows them to establish cation-π interactions with the positively charged quaternary ammonium lipid headgroups [41], [43]. At low pH, the optimal pH for HCV fusion, these interactions would be favored due to the protonation of the amino groups. As was described for other substituted indoles [44], it is possible that protonation of the carbon bearing the ester group of Arb could displace this group out of the indole plane, and place it in a better position to bond with neighboring molecules. This could in turn lead to a better membrane association. Arb might therefore have the propensity to intercalate into lipids of the viral and target membranes while adopting a consistent orientation by filling the gaps between lipid molecules. The interfacial region of the lipid bilayer provides a suitable environment for a wide range of chemical groups, as long as they possess a large enough hydrophobic moiety and a group capable of forming hydrogen bonds with the lipid carbonyl groups. Several compounds with antiviral pharmacological properties belong to this category, in particular adamantanes active against influenza A viruses [45], [46] and against some HCV clones but not all [47], [48], the natural triterpene glycyrrhizin efficient in the treatment of chronic viral hepatites [49] and the flavonolignan molecules composing silymarin, an herbal extract with potent anti-HCV activities [1]–[3], [50], [51].

In a previous study, we noticed that Arb inhibition of cell entry concerned HCVpp and pseudoparticles bearing the influenza hemagglutinin (HApp), but not pseudoparticles bearing the envelope glycoprotein of a feline oncogenic retrovirus (Rd114pp) [12]. These data suggest that Arb might display selectivity for the recognition of key motifs inside envelope proteins. This hypothesis was tested by assessing the influence of Arb on the fluorescence properties of aromatic compounds derived from tryptophan (Trp) and of peptides containing Trp. Trp is a component of proteins with interfacial properties [41], [42], often located at the lipid/water interface and grouped into so-called tryptophan-rich motifs crucial to protein/membrane association [52], and found in the envelope (fusion) proteins of the SARS coronavirus or HIV-1 [36], [53], [54]. Trp is also enriched at protein/protein binding interfaces of the small envelope protein of the hepatitis B virus [55] and of membrane proteins in general [56]. We demonstrated here that Arb was able to alter/quench the fluorescence properties of small Trp derivatives in solution (NATA), in detergent micelles and in liposomes (TOE, [57]), in a dose-dependent manner. This occurred most likely through stacking of the aromatic rings of both molecules which is often involved in stabilization of inter-cations. Interestingly the apparent affinity of the Arb/Trp derivative interaction was in the order: lipid bilayers>micelles>solution, indicating that Arb binding strength for Trp could increase in membrane environments where both molecules accomodate and get packed. Indeed Arb apparent affinity for TOE in liposomal membranes was in the 10 µM range, a value comparable to the IC50 of fusion. Arb affinity was even greater for membrane peptides containing Trp and tyrosine (Tyr) residues (ca. 4 µM). Due to its indole group, it is conceivable that Arb might display selectivity not only for indole rings (Trp) but more generally for aromatic groups, as the phenol ring of Tyr. A greater number of Arb molecules could therefore interact with aromatic residues in peptide sequences, leading to some cooperativity in the quenching effect and to an overall larger apparent affinity.

Although HCV entry inhibition by Arb was found genotype-dependent, HCV membrane fusion was inhibited by Arb in a genotype-independent manner. HCV entry and fusion are early steps in the life cycle of the virus [58], [59]. HCV first interacts through its envelope glycoproteins with a set of coreceptors at the plasma membrane level (recently reviewed in [60], [61]) and eventually becomes endocytosed [62]–[64]. Due to a combined action of acidification in the endosome and particular lipids like cholesterol and sphingomyelin [11], [18], viral fusion occurs over a broad spectrum of pH's ranging from 6.3 to 5.0 [11], [18], [65]. HCV binding to the hepatocyte membrane followed by endocytosis therefore requires several cellular proteins, and most likely involves several levels of interactions (interactions between viral proteins, between cellular and viral proteins, between viral/cellular proteins and lipids). These features might explain the differential effect exerted by Arb on entry of various HCV genotypes: indeed subtle differences in protein sequences could translate into modified interactions with several partners and/or at several levels. Conversely some common principles of action apply to all fusion reactions, viral fusion and cellular fusion processes alike [66]. Indeed all fusion processes involve two partners: lipids and the fusion protein(s). This might account for the similar inhibitory effect of Arb on HCV fusion observed for all genotypes. This is in line with the observations that Arb displayed potent antiviral activity against some antigenic serotypes of influenza viruses, but not against all [9].

Previously we noticed that Arb inhibition of primary infection of Huh-7.5.1 cells with HCV (clone JFH-1) was efficient only when cells were preincubated with Arb 24 or 48h before infection [10]; in addition, inhibition of HCVpp and HApp cell entry was most efficient when Arb was pre-incubated with both viral and cell membranes [12]. Here, using our *in vitro* fusion assay, we observed that Arb inhibition of HCVpp fusion was maximal when both viral and target membranes were incubated with Arb, before fusion was initiated. This suggests that a certain level of membrane impregnation and/or saturation with Arb must be achieved to efficiently inhibit viral infection. Membranes might therefore act as “concentrators” of arbidol, and high concentrations of the molecule might be locally achieved. This could explain why Arb, exhibiting an apparent (medium to low) affinity for membranes in the µM range, exerts a relevant antiviral activity without noticeable membrane damages. Along these lines, in spite of its marked membranotropism, Arb displays only low toxicity [9], [10]. Arb exhibited a comparable micromolar apparent affinity for aromatic residues present in membrane peptides in a membrane environment. Altogether, these observations lead us to propose a mechanistic model of the way Arb would inhibit HCV entry and fusion. Through its membranotropism, Arb is able to freely interact with viral and target membranes, and could locally get highly concentrated. Arb is also able to interact with aromatic residues within viral proteins involved in membrane interactions and membrane destabilization necessary for fusion. Through this dual binding capacity, Arb could then ***locally impede*** the structural rearrangements required for the fusion protein to adopt its fusion conformation. The fact that Arb is active in the µM range suggests that Arb would act by reducing the overall speed of the fusion reaction rather than by blocking a specific protein conformation. This could therefore explain the broad antiviral spectrum of Arb, and the genotype independence of its inhibitory effect on HCV fusion, since HCV envelope proteins contain well-conserved aromatic residues in all genotypes. Mechanistically, the key point is the relative accessibility of these residues to Arb at the membrane interface. A cooperative effect between Arb and several aromatic residues might therefore occur. Also the local environment of these aromatic aa is important, since the presence of residues such as histidines (His) in their vicinity could modify their accessibility with respect to pH. Interestingly enough, in the sequence of both HCV E2 peptides studied here (Table 5) and shown to be involved in HCV fusion [17], His is contiguous to Trp, and in the 606–625 peptide, His is surrounded by three tyrosines. The concept of His as a critical pH sensor at a key intramolecular domain interface in a viral fusion protein has recently emerged [67], [68]. Indeed, the protonation of a sole His in the E protein of the tick-borne encephalitis flavivirus (TBEV) triggers large-scale conformational changes leading to viral fusion. Concerning HCV, Rey and coworkers recently proposed a model of the 3D arrangement of the E2 ectodomain [39]. In this model, the fusion loop/peptide would lie within the poorly structured domain II, and the E2 606–625 peptide would be found in the globally unstructured domain III, where a critical His residue is disposed at the interface with domain I. The putative fusion loop contains a phenylalanine and a tyrosine [39]. At low pH, the optimal pH for HCV membrane fusion, key histidine(s) could become protonated. This could result in conformational rearrangements and, in the context of Arb fusion inhibition, aromatic residues might consequently become more or less accessible to Arb molecules present in their vicinity. We noted that the apparent affinity of Arb for HCV peptides was weaker at pH 5.0 than at pH 7.4. At low pH, Arb is also protonated, and this protonated form could exhibit a greater preference for the interfacial region of the lipid bilayer than the deprotonated form, as demonstrated for adamantanes [45]. Combined with the notion that key aromatic and His residues would also display interfacial (re)localization at low pH, this would in turn explain the higher efficiency of Arb at inhibiting fusion at acidic pH [12].

In conclusion our data reveal that Arb directly interacts with the lipid membrane-water interface, and is able to bind to aromatic residues present in HCV glycoproteins, in their membrane-associated form. Through a subtle binding interplay between Arb, lipids, viral and cellular proteins, Arb might efficiently block HCV entry and membrane fusion interacting with the main actors of the early steps of viral entry. Most interestingly, Arb inhibition of these processes demonstrated an affinity in the µM range, although the membranotropic properties of Arb suggest that it could become locally more concentrated in membranes. Together, these findings suggest that Arb could increase the strength of viral glycoprotein's interactions with the membrane due to a dual binding mode, involving aromatic residues and phospholipids. The resulting complexation would inhibit the expected viral glycoprotein conformational changes required during the membrane fusion process.

The antiviral mechanism of Arb therefore opens promising perspectives for the development of small membranotropic low affinity molecules, that would become locally concentrated in membranes and would mainly act on the kinetics of the conformational rearrangements of the viral fusion protein.

## Supporting Information

Figure S1 **Arb inhibits infectivity and membrane fusion in a dose-dependent manner.** A, Infectivity. Results are the mean ± SEM of 5 separate experiments. Black, no Arb; blue, 1.9 µM; green, 5.6 µM and red, 11.3 µM Arb, respectively. B, Membrane fusion between HCVpp of genotype 4 (4.11.21) and R18-labeled liposomes. The lipid mixing kinetic was followed by fluorescence spectroscopy using excitation and emission at 560 and 590 nm, respectively. Fluorescent liposomes (12.5 µM final lipid concentration) were added to 40 µl of HCVpp in PBS pH 7.4 at 37°C, in the absence or presence of the indicated concentrations of Arb. After a 2 min-equilibration, lipid mixing was initiated by decreasing the pH to 5.0 with diluted HCl, and R18 dequenching was recorded. Maximal fluorescence was obtained after addition of 0.1% final Triton X-100. Average value of the last 30 s of fusion (i.e. final extent of fusion) was used to calculate the percentage of fusion in the presence of Arb, relative to 100% fusion without Arb (Figure 2). Black, no Arb; blue, 1.9 µM green, 5.6 µM and red, 11.3 µM Arb, respectively.
(TIF)

Figure S2 **NMR of Arbidol into lipid bicelles.** A, 1H/13C HSQC spectrum; [Arb]/[lipid] ratio was 1/15 and temperature 305 K. B, extract of 1H NOESY spectrum; [Arb]/[lipid] ratio was 1/10, temperature 290 K and mixing time 200 ms.
(TIFF)

Figure S3 **Trp emission fluorescence spectrum of a BVDV model peptide into PC∶chol liposomes.** BVDV NS5A peptide (SGNYVLDLIYSLHKQINRGLKKIVLGWA, 5 µM final) reconstituted into PC∶chol liposomes (70∶30 molar ratio, peptide-to-lipid ratio 1∶20) were equilibrated to 37°C in PBS at pH 7.4 or 5.0 in the absence (thick line) or presence (thin lines) of increasing concentrations of Arb (2, 5, 10, 25 and 100 µM from top to bottom). Trp emission fluorescence was measured between 300 and 400 nm, with excitation at 286 nm. The apparent affinity of Arb toward Trp was calculated from the plot of the difference ΔA between areas under the curve (AUC) of peptide without Arb (ΔA = AUC_no Arb_−AUC_with Arb_) as a function of Arb concentration (insert).
(TIF)
