# Supplementary figures and images for: Mechanism of Inhibition of Enveloped Virus Membrane Fusion by the Antiviral Drug Arbidol
Source: PLoS One. 2011 Jan 25;6(1):e15874. doi: 10.1371/journal.pone.0015874 (PMC3026800; doi:10.1371/journal.pone.0015874)

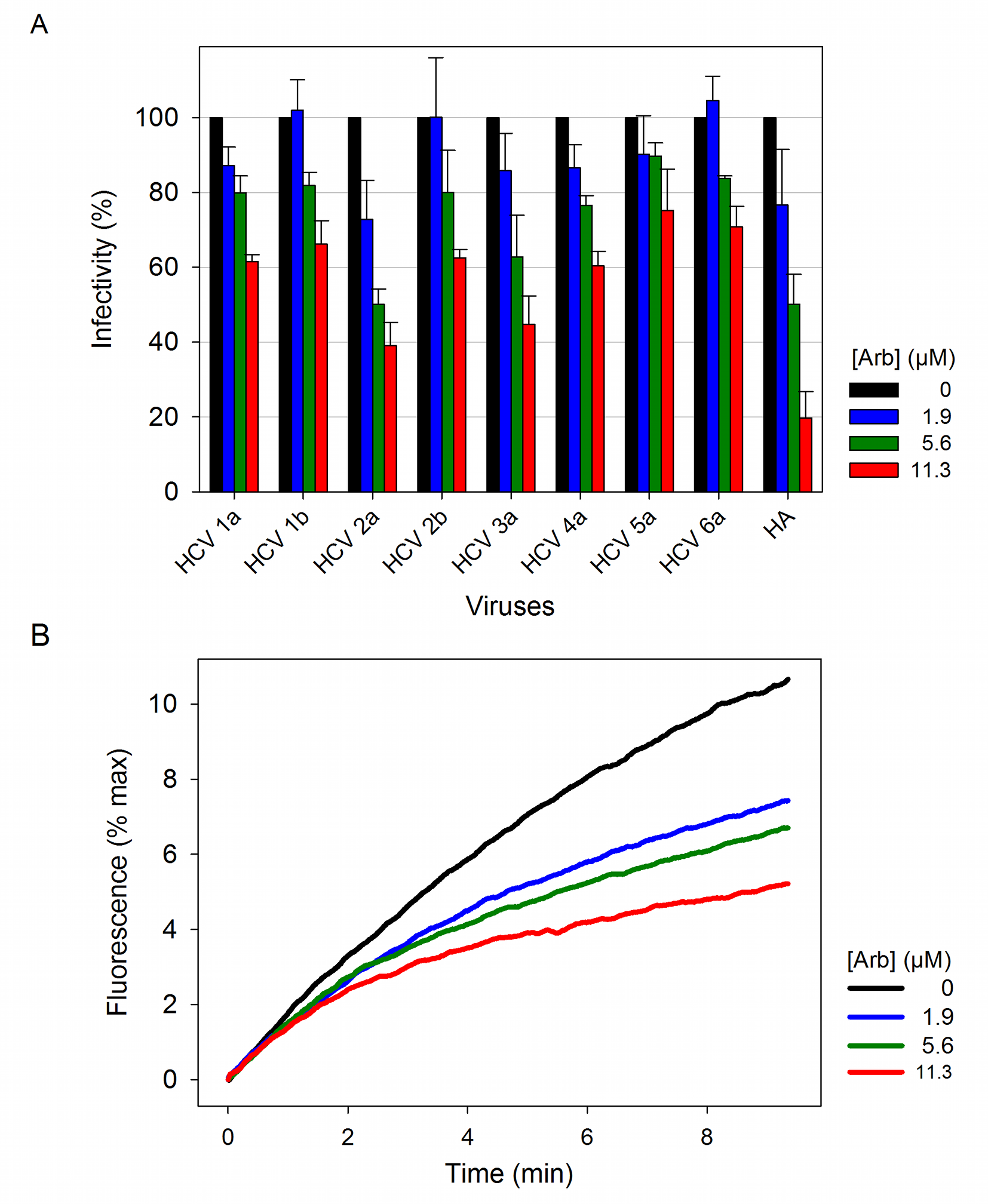

Supplement: Figure S1 — Arb inhibits infectivity and membrane fusion in a dose-dependent manner. A, Infectivity. Results are the mean ± SEM of 5 separate experiments. Black, no Arb; blue, 1.9 µM; green, 5.6 µM and red, 11.3 µM Arb, respectively. B, Membrane fusion between HCVpp of genotype 4 (4.11.21) and R18-labeled liposomes. The lipid mixing kinetic was followed by fluorescence spectroscopy using excitation and emission at 560 and 590 nm, respectively. Fluorescent liposomes (12.5 µM final lipid concentration) were added to 40 µl of HCVpp in PBS pH 7.4 at 37°C, in the absence or presence of the indicated concentrations of Arb. After a 2 min-equilibration, lipid mixing was initiated by decreasing the pH to 5.0 with diluted HCl, and R18 dequenching was recorded. Maximal fluorescence was obtained after addition of 0.1% final Triton X-100. Average value of the last 30 s of fusion (i.e. final extent of fusion) was used to calculate the percentage of fusion in the presence of Arb, relative to 100% fusion without Arb (Figure 2). Black, no Arb; blue, 1.9 µM green, 5.6 µM and red, 11.3 µM Arb, respectively. (TIF) [file pone.0015874.s001.tif]

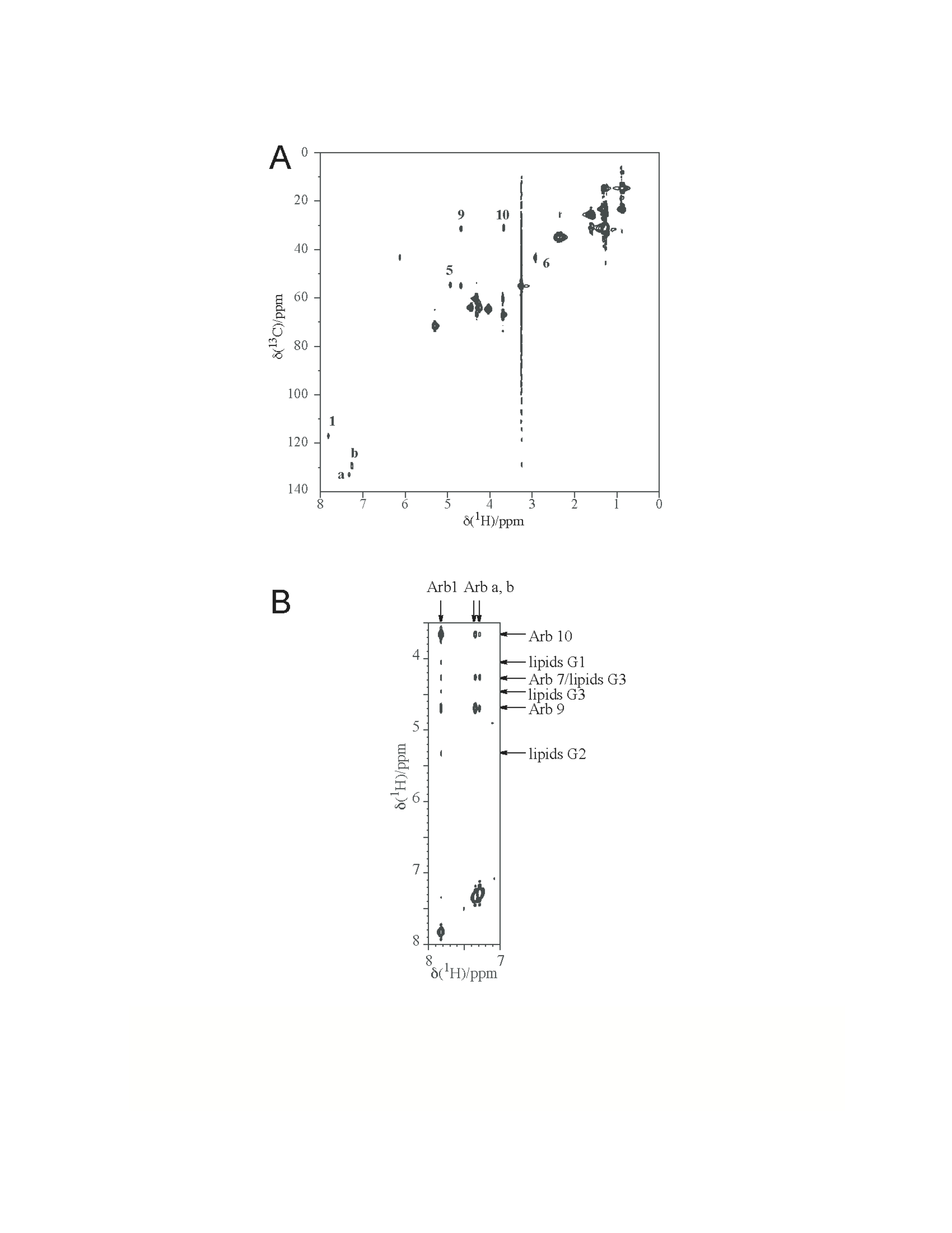

Supplement: Figure S2 — NMR of Arbidol into lipid bicelles. A, 1H/13C HSQC spectrum; [Arb]/[lipid] ratio was 1/15 and temperature 305 K. B, extract of 1H NOESY spectrum; [Arb]/[lipid] ratio was 1/10, temperature 290 K and mixing time 200 ms. (TIFF) [file pone.0015874.s002.tif]
